# Screening and identification of seed-specific genes using digital differential display tools combined with microarray data from common wheat

**DOI:** 10.1186/1471-2164-12-513

**Published:** 2011-10-17

**Authors:** Xinglu Yang, Hongliang Xu, Wenhui Li, Le Li, Jinyue Sun, Yaxuan Li, Yueming Yan, Yingkao Hu

**Affiliations:** 1 College of Life Sciences, Capital Normal University, Beijing, 100048, China; 2 Plant Biotechnology Institute, National Research Council Canada, Saskatoon, S7N 0W9, Canada

## Abstract

**Background:**

Wheat is one of the most important cereal crops for human beings, with seeds being the tissue of highly economic value. Various morphogenetic and metabolic processes are exclusively associated with seed maturation. The goal of this study was to screen and identify genes specifically expressed in the developing seed of wheat with an integrative utilization of digital differential display (DDD) and available online microarray databases.

**Results:**

A total of 201 unigenes were identified as the results of DDD screening and microarray database searching. The expressions of 6 of these were shown to be seed-specific by qRT-PCR analysis. Further GO enrichment analysis indicated that seed-specific genes were mainly associated with defense response, response to stress, multi-organism process, pathogenesis, extracellular region, nutrient reservoir activity, enzyme inhibitor activity, antioxidant activity and oxidoreductase activity. A comparison of this set of genes with the rice (*Oryza sativa*) genome was also performed and approximately three-fifths of them have rice counterparts. Between the counterparts, around 63% showed similar expression patterns according to the microarray data.

**Conclusions:**

In conclusion, the DDD screening combined with microarray data analysis is an effective strategy for the identification of seed-specific expressed genes in wheat. These seed-specific genes screened during this study will provide valuable information for further studies about the functions of these genes in wheat.

## Background

As one of the most important crops in the world, the different processing characters of wheat such as milling, baking, and cooking are largely determined by the biochemical composition of the seeds [1]. The nutritional profile of the seed is determined by the physiological, biochemical and morphological changes during grain development. Some morphogenetic and metabolic processes are exclusively associated with seed maturation. All these fundamental processes are delicately regulated at the level of transcription. Therefore, it will be necessary to reveal the underlying molecular mechanisms determining grain quality [2,3], especially those genes whose expression patterns are seed-specific.

The expression level of a candidate gene is commonly estimated using two analysis approaches referred to as 'analog' and 'digital' [4]. The analog approach is based on oligonucleotide probe hybridizations such as Northern blotting, mRNA differential display, and DNA microarrays, while the digital approach is based on high-throughput generation of gene transcripts as in the case of Expressed Sequence Tags (ESTs).

cDNA microarrays, corresponding to the analog approach, are used to provide a comprehensive description of transcript level in an organism after perturbation or during development [5]. They have been widely used in studying the biological processes during grain growth and filling in cereals [3,6-8]. For wheat they have also been extensively used to analyze the expression of genes involved in the development of seed [3,9-11]. However, not all labs have the ability to perform microarray analysis. The publicly available microarray resources allow scientists to gain comprehensive information and knowledge of gene expression profiles in particular tissues and organs at different developmental stages.

DDD corresponding to the digital approach is an online bioinformatics tool for identification of differentially expressed genes based on the relative abundance of ESTs from various libraries, or pools of libraries, represented in UniGene [12]. To eliminate the difference caused by the unequal number of ESTs in each selected libraries, DDD uses the Fisher Exact Test [13] to restrict the output for statistically significant differences (P ≤ 0.05). This approach has proven to be very useful in the study of organ-specific genes in mammal [14] and cancer cells [15]. Despite these works, some work of *in silico *expression studies have been conducted in wheat [16,17]. The deposition of the generated gene transcripts in public databases provides a valuable tool for *in silico *analysis of organ specific expressed genes.

Determination of genes involved in seed development and their functions is one of the major goals in plant developmental biology. In the current work, genes specifically expressed in wheat seeds were screened and identified by using DDD, together with the available online microarray databases. These wheat seed-specific genes were also compared with rice genome to examine whether the expression patterns of homology gene groups involved in seed development are conserved between wheat and rice.

## Results

### DDD

**Figure 1:**
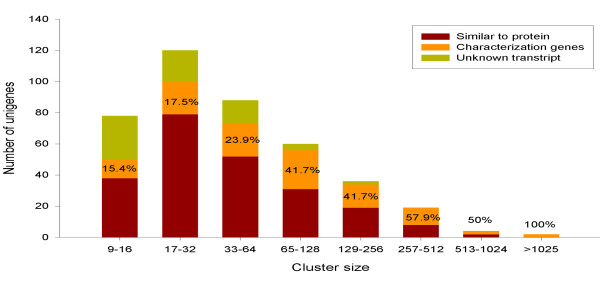
**407 unigenes based on DDD selection sorted by cluster-size**. The number of unigenes in each size-based cluster bins was plotted. The ration of CG gene models in each size-based was shown.

DDD comparison between seed libraries and non-seed libraries was used to identify unigene sets that were seed-specific. The libraries chosen for comparison were released on July 19, 2010 (Table 1 and Additional file 1). Following the formula mentioned in the methods, four hundred and seven unigene sets differentially expressed in selected libraries were retrieved. Among them, 108 (27%) unigenes were characterized gene models (CG), and 228 (56%) unigenes had no corresponding protein of known function but show similarity (identity > 45%) to proteins of other species (SG). In addition, the other 71 (17%) unigenes represented unknown transcripts gene models (UG). According to cluster sizes (the sum of ESTs), their expression abundance was classified into different levels, each level contains various amount of unigenes (Figure 1). From figure 1, it is clear that the ratio of the CG gene models is high when the size of unigene is large. In other words, it is more likely to characterize the unigene when the cluster size is large.

**Table 1:** Summary of wheat libraries used in the study

	Pool A			Pool B	
Seed organs	Libraries	ESTs	Non-seed organs	Libraries	ESTs
Endosperm	5	11346	Callus	1	9685
Seed embryo	2	4463	Crown	4	14529
Seed	8	67394	Leaf	25	76992
Kernel	4	13850	Shoot	12	94514
Grain	9	54579	Root	16	169382
Total	28	151632	Seedlings	2	2305
			Heads	4	5143
			Liquid cultured tissue	1	10164
			Sheath	1	1068
			Spike	2	26307
			Floral organs	12	60963
			Young spikelets	1	2093
			Total	81	473145

The details for the libraries were listed in additional file 1.

### Identification of genes specifically expressed in seeds using microarray data

**Figure 2:**
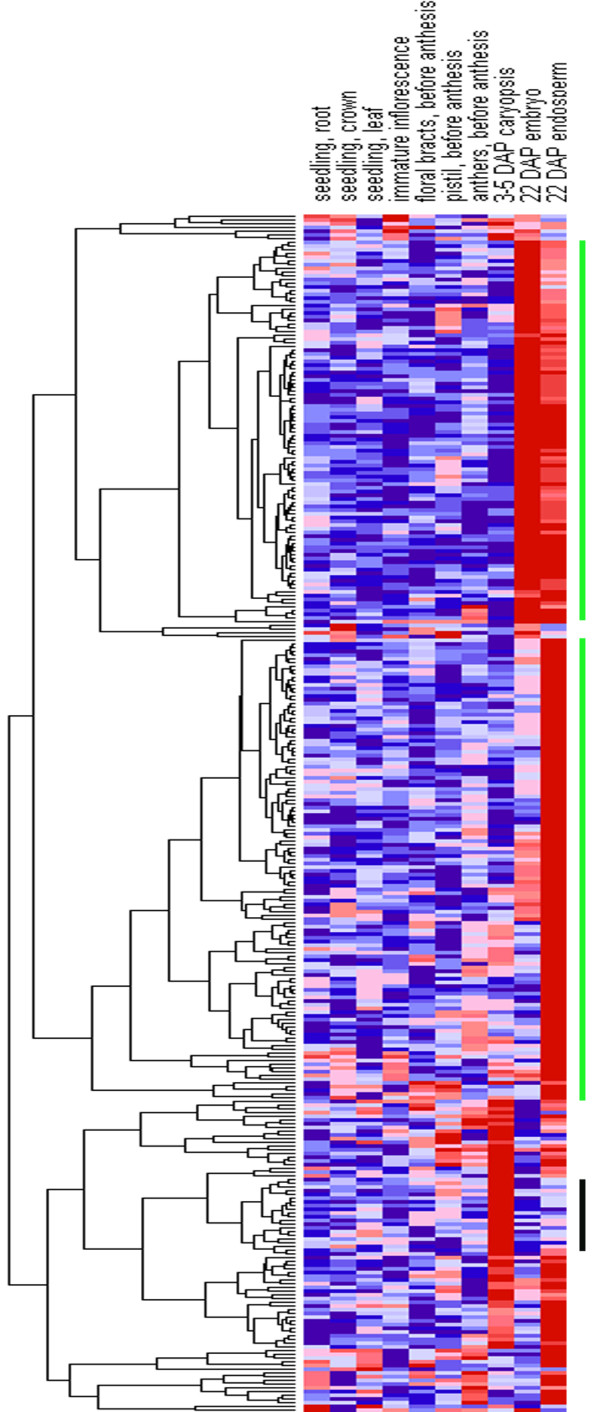
**A hierarchical clustering of the 322 probesets according to their expression patterns in different organs**. The 322 probesets correspond to 256 unigenes screened by DDD. The hierarchical cluster color code: the largest values are displayed as the reddest (hot), the smallest values are displayed as the bluest (cool), and the intermediate values are a lighter color of either blue or red. Pearson correlation clustering was used to group the developmentally regulated genes. The vertical green bars on the right mark the genes which show specificity in the 22 DAP caryopsis while the black one marks the genes which show specificity in the 3-5 DAP caryopsis.

Based on extensive EST collections, Affymetrix GeneChip platforms have now been developed for wheat [18]. The microarray data for the genes screened above with DDD were searched against PLEXdb (http://www.plexdb.org) [19]. A total of 322 probesets corresponding to 256 (63%) unigenes were analyzed here. To better assess the data, a heat map was generated through the GenePattern program (http://www.broadinstitute.org/cancer/software/genepattern/index.html) [20] (Figure 2). Figure 2 indicated that not all probesets showed specific expression in seeds. The probesets with expression levels significantly higher in seeds than any other organs (more than 4-fold) were chosen for further analysis. Finally, 236 probesets corresponding to 201 unigenes were selected (Additional file 2). Among them, 47 (23%) were CG gene models, 119 (59%) were SG gene models and the other 35 (17%) were UG gene models. Furthermore, among the 201 unigenes, most (173 unigenes) showed specificity in the 22 DPA caryopsis (embryo and endosperm), while 14 unigenes showed specificity in the 3-5 DPA caryopsis.

Microarray-alone analysis was also performed to screen probsets corresponding to genes with seed-specific expression. The 236 probsets screened above were used as "seeds" to find seed-specific genes in wheat microarrays. The searching tool was "Profile Neighbors" in PLEXdb (http://www.plexdb.org//modules/glSuite/gl_main.php) [19]. One hundred and seventy-four additional probsets were retrieved by removing the redundant information and probsets with expression profiles that were not seed-specific. The EST profiles were also screened for the unigenes corresponding to the 174 probesets. However, 95 of the corresponding unigenes were not for seed-specific ESTs.

### GO enrichment analysis

**Figure 3:**
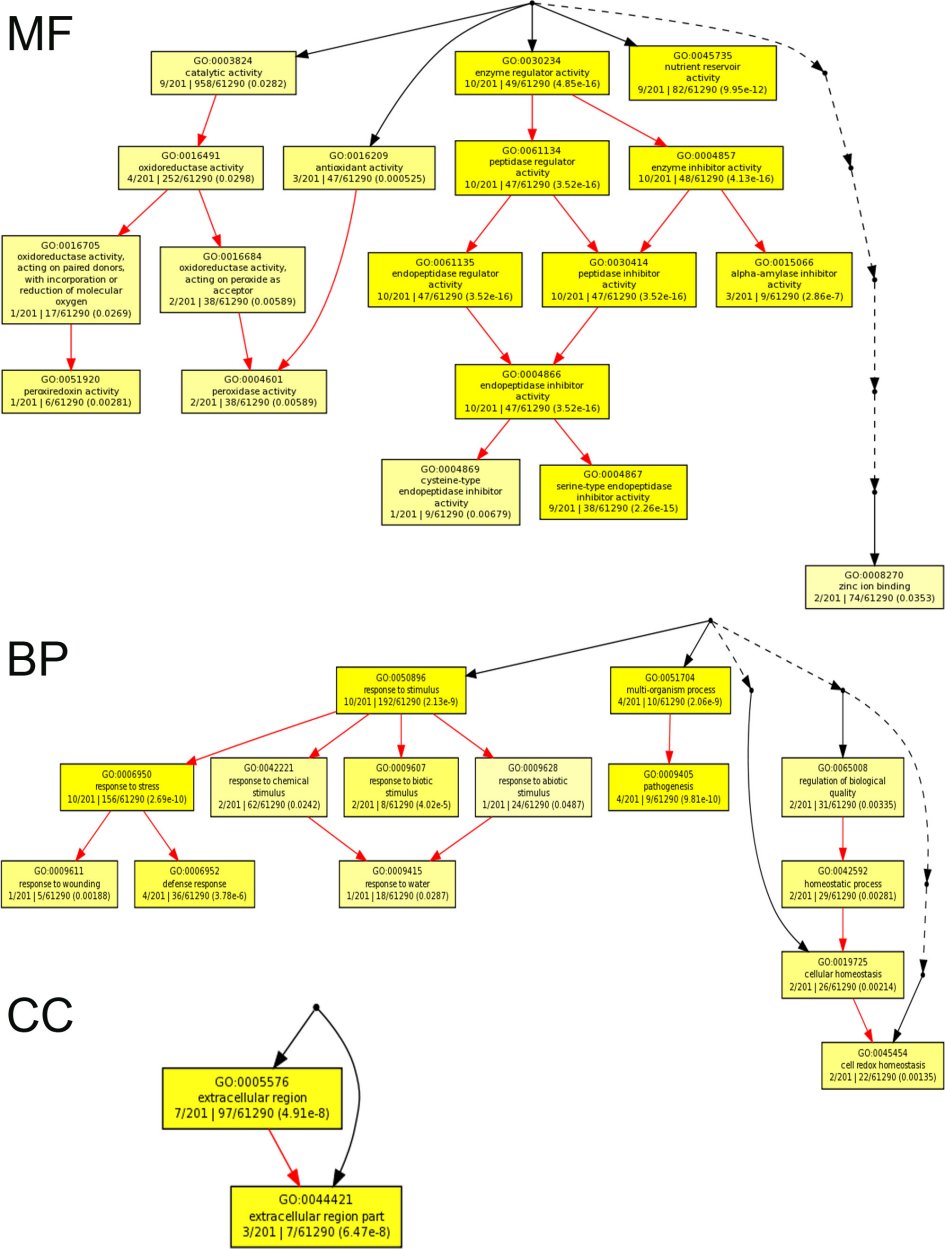
**GO enrichment analysis of 201 seed-specific genes in wheat**. The graph displays enriched GOIDs of 201 seed-specific genes and their hierarchical relationships in "biological process (BP)", "cellular component (CC)" or "molecular function (MF)" GO categories. Boxes represent GO terms, term definition, p-value and detail information. Significantly enriched GO terms are marked yellow. The degree of color saturation of each node is positively correlated with the significance of enrichment of the corresponding GO term. Non-significant GO terms within the hierarcical tree are shown as points. Branches of the GO hierarchical tree without significant enriched GO terms are not shown. Edges stand for connections between different GO terms. Red edges stand for relationship between two enriched GO terms, black solid edges stand for relationship between enriched and unenriched terms, black dashed edges stand for relationship between two unenriched GO terms.

Further GO enrichment analysis was performed for the 201 seed-specific unigenes through GOEAST (Figure 3). GOEAST is a web based software providing easy to use, visualizable, comprehensive and unbiased Gene Ontology (GO) analysis (http://omicslab.genetics.ac.cn/GOEAST/index.php) [21]. The details of the enrichment are listed in table (Additional file 3). Most of the unigenes were assigned into several categories, including stress response, defense response, multi-organism process, pathogenesis, extracellular region, nutrient reservoir activity, enzyme inhibitor activity, antioxidant activity and oxidoreductase activity (Figure 3).

According to the expression specificity analysis results, 14 of 201 unigenes were specially expressed in the 3-5 DPA caryopsis. At 3-5 DPA, the caryopsis is at the coenocytic stage during which free nuclear division occurs within the primary endosperm cell to give a coenocytes. In wheat, over 2000 nuclei will be generated within 72 hours after fertilization [22]. During this stage the seed is far from mature and has not begun accumulating grain storage molecules. This explains why the 14 unigenes whose expressions were specific in the 3-5 DPA caryopsis didn't contain any storage protein transcripts. Meanwhile, 173 (86%) unigenes were found to be specifically expressed in the 22 DPA caryopsis. During the grain filling stage (14-24 DPA) the major metabolic activity is the synthesis and accumulation of storage molecules such as starch and protein. Therefore, the genes belonging to the "nutrient reservoir" category all represented in this stage. Ten genes, which have specificity in the 22 DPA caryopsis, were found in the category of "response to stress". This is consistent with the result of Becerra et al. [23] who found 6 genes specially expressed in the Arabidopsis seed that were involved in response to abiotic stress, which clearly indicated the importance of genes contributing to stress-tolerance during seed development. Interestingly, there are also 9 genes with function annotation as "serine-type endopeptidase inhibitor activity" showed specific expression in the 22 DPA caryopsis. Three genes of this category encode serpins, which are inhibitors of exogenous proteinases (capable of breaking down seed storage proteins); play important roles in the defence of specific cell types of vegetative tissues [24,25]. Another three genes encode alpha-amylase inhibitor which plays a similar role as protease inhibitors and protect cereal seed from the attack by endogenous hydrolase [26]. Thus, during the filling stage, in addition to transcript accumulation of genes encoding storage protein, genes involved in protecting the main nutritional resource of the developing seeds from endogenous as well as exogenous attack are also seed-specific and active.

### The expression patterns of homologous gene groups in rice

Both rice and wheat belong to the plant family Gramineae and rice is the best-characterized experimental model for monocot plants. Further more, these two species are evolutionarily related. To investigate whether the expression patterns of homologous gene groups involved in seed development are conserved between wheat and rice, the 201 unigenes which showed seed-specific in wheat were compared with homologous genes in rice. For some unigenes, no significant homology was found in the rice genome, while for others, more than one unigene corresponded to the same homologous gene in rice. In the end, 121 (60%) unigenes corresponding to 106 putative homologous genes in rice were inferred based on sequence similarity. To facilitate the comparison of 106 homologous genes between wheat and rice, they were divided into three categories as follows: 13 gene models with high similarity (HS: >90% in the aligned region), 52 gene models with moderate similarity (MS: 70-90% in the aligned region) and 41 gene models with weak similarity (WS: 45-70% in the aligned region) to their counterpart genes.

**Figure 4:**
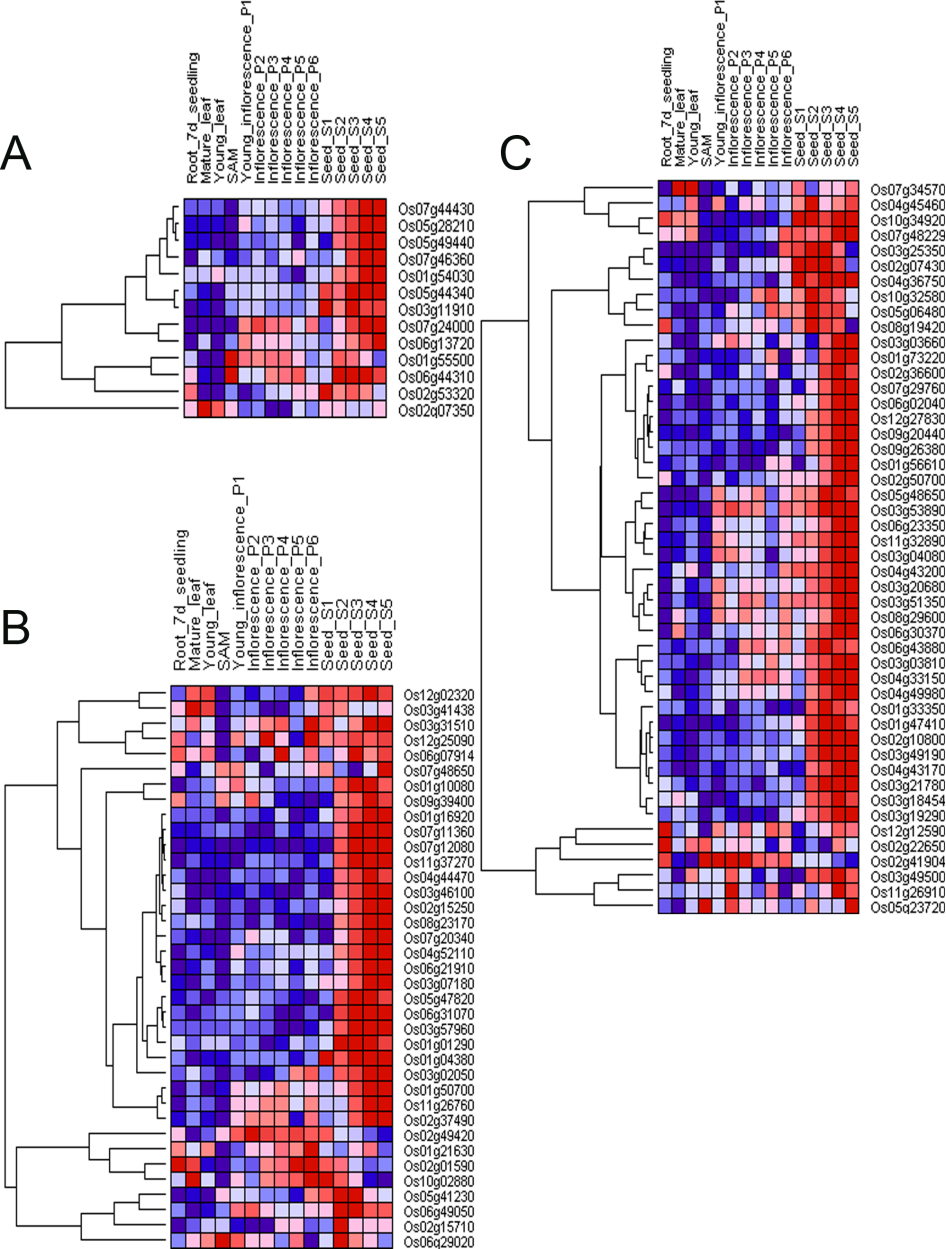
**Homologous genes of rice grouped in clusters according to their expression patterns in different organs**. (A) 13 of 98 genes show high similarity (B) 37 of 98 genes show weak similarity (C) 48 of 98 genes show moderate similarity. The accession number of each gene was listed on the right. The hierarchical cluster color code: the largest values are displayed as the reddest (hot), the smallest values are displayed as the bluest (cool), and the intermediate values are a lighter color of either blue or red. Pearson correlation clustering was used to group the developmentally regulated genes.

To examine whether the three gene models have a seed-specific expression pattern in rice, the IDs of probesets representing the homologous genes and the microarray data presenting on the Affymetrix rice genome array were retrieved using an online tool of internet based database - Rice Multi-Platform Microarray Search tool (http://www.ricearray.org/matrix.search.shtml) [27]. A total 98 homologous sequences were detected, while 8 of the 106 homologous sequences had no corresponding probe on the rice arrays. Hierarchical clustering analysis based on array data had also been carried out (Figure 4). The results indicate that the expression patterns of homologous gene groups are conserved at different degrees between wheat and rice. Though not all the analyzed genes showed seed-specificity in rice, a large percentage of corresponding genes in each gene models were specially expressed in the seed of rice. Genes are defined as seed-specific only if their expression levels in the seed is significant higher (more than 4-fold) than that in any other organs. Finally, the rice counterparts of 9 out of 13 (69%) HS gene models, 31 out of 48 (65%) MS gene models, and 23 out of 37 (62%) WS gene models were all found to be seed-specific. The results also indicated that the percentage of seed-specific genes among HS, MS and WS gene models both differed and were lower where less sequence similarity occurs.

In order to assess the quality of the list, we also performed a sensitivity analysis. Seventy genes were identified as seed-specific in rice by the same method (DDD combined the microarray) which was used in wheat. Meanwhile, microarray-alone analysis in rice was also performed using the same method applied in wheat and 358 probsets were screened as seed-specific genes. The libraries chosen for DDD comparison were released on Apr 5, 2011 (Additional file 1). The 70 seed-specific genes in rice were compared with the genome of wheat and 43 (61%) homologous genes were found. Among the 43 homologous genes in wheat, 27 were seed-specific and detected in the list of 201 wheat seed-specific unigenes screened here.

As for the various datasets of seed-specific genes screened by DDD-alone, microarray-alone and DDD + microarray, the qualities of these lists were all evaluated by the specificity (% of wheat seed-specific genes replicating in rice) and sensitivity (% of rice seed-specific genes replicating in wheat) analysis (Table 2). It is clear that both specificity and sensitivity were higher in DDD + microarray analysis than any single analysis. And the number of replicate genes does not drastically reduce in the DDD + microarray comparing to the other two lists.

**Table 2:** The specificity and sensitivity analysis of various seed-specific lists

	Spec(%)	Sens(%)
DDD-alone	7.6	27
Microarray-alone	6.6	11.7
DDD + Microarray	11.4	38.5

Spec = % of wheat seed genes replicating in rice, Sens = % of rice seed-specific genes replicating in wheat. The amount of genes of each seed-specific gene list is 407 for DDD-alone, 410 for microarray-alone and 201 for DDD + microarray in wheat; 111 for DDD-alone, 358 for microarray-alone and 70 for DDD + microarray in rice.

GO enrichment analysis was also performed for the 70 seed-specific genes in rice through the GOEAST [21] (Additional file 4). The result showed that most rice seed-specific genes were also associated with oxidation-reduction process, response to stress, defense response, envelope, extracellular region, nutrient reservoir activity and enzyme inhibitor activity. These results suggest that most of the seed-special genes in both wheat and rice have shared functions.

### Experimental validation of the expression patterns of 10 selected unigenes by qRT-PCR

**Figure 5:**
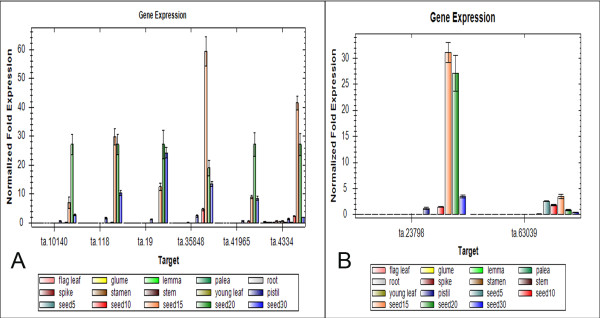
**qRT-PCR analysis of the expression profiles of eight genes selected from *in silico *screening results**. (A) Six unigenes isolated by the combination of DDD selection and microarray data analysis. (B) Two unigenes isolated only by DDD selection. The x-axis of each gene represents for different tissues or organs. The bars above each gene name indicate different tissues or organs with a single color. The order from left to right is: flag leaf, glume, lemma, palea, root, spike, stamen, stem, young leaf, pistil, seed5, seed10, seed15, seed20 and seed30. The y-axis shows the gene expression levels after normalization to reference genes Ta.54227 and Ta.2291.

**Figure 6:**
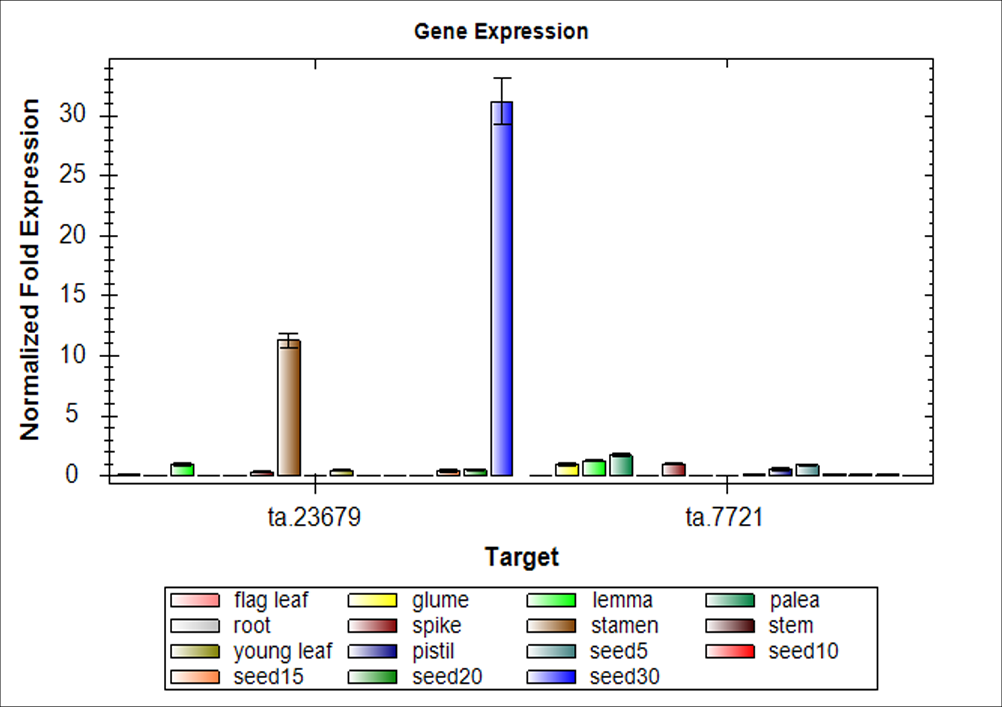
**qRT-PCR analysis of two genes which didn't meet the formula in the selection of DDD**. The bars above each gene name indicate different tissues or organs with a single color. The order from left to right is: flag leaf, glume, lemma, palea, root, spike, stamen, stem, young leaf, pistil, seed5, seed10, seed15, seed20 and seed30. The y-axis shows the gene expression levels after normalization to reference genes ta.54227 and ta.2291

To confirm the results of DDD and microarray screening, ten unignes were selected for validation of their expression specificity with quantitative real-time PCR (qRT-PCR). Two transcripts (Ta.54227 Ta.2291), which were previously shown to have consistent expression across samples, were chosen as internal controls for all the data normalization [28]. Among the 10 genes, 6 were randomly selected from the CG gene models generated by both DDD and microarray selection (Figure 5A), two were randomly selected from the group generated by DDD based selection but without microarray data (Figure 5B). All of the eight genes tested by qRT-PCR showed the same seed specificity as *in silico *analysis. Meanwhile, the other two genes which didn't follow the formula used in the DDD selection, that is the rations in poolB were indicated as <0.00005 while the sum of ESTs in poolA is less than 4 times than that in poolB, were also selected for qRT-PCR analysis (Figure 6). The results showed that none of the two genes showed seed specificity.

## Discussion

This study demonstrates that DDD combined with microarray data, is an effective method to identify and analyze genes specifically expressed in the developing seed of wheat. Becerra et al. [23] used EST virtual subtraction combined with microarray data analysis to discover Arabidopsis genes specifically expressed in immature seeds. Eujayl et al. [16] identified differentially expressed unigenes in developing wheat seeds of various species and different stages using only DDD analysis. In Eujayl's study, apart from seed storage genes, other 46 unigenes were identified as seed-specific, although, only 23 unigenes were described in their report. Among the 23 unigenes which were reported, 10 were included in our result of 201 unigenes. As for the remaining part, 3 unigenes had been retired from the database, 5 had no microarray data and the other 5 unigenes were shown not to be seed-specific by the microarray data.

The expression level of a gene is commonly estimated using two analysis approaches referred to as 'analog' and 'digital'. To identify seed-specific genes in wheat, both of these approaches were used in our study. Among the 407 unigenes preliminarily screened by DDD, there are 33 CG gene models encoding the wheat storage proteins such as gliadin, glutentin, triticin and avenin. There are also two other CG gene models encoding late embryogenesis abundant (LEA) proteins which are the most abundantly expressed proteins in the seeds. Since the accumulation of seed storage proteins and LEA proteins are both highly seed-specific processes [29], the coverage of these genes with known tissue specificity demonstrates the feasibility of DDD methods in wheat. However, because of the limitation of the quantity and diversity of ESTs in wheat, the results of single DDD screening were not entirely accurate. In our study, 55 unigenes were proved to be not seed-specific by microarray analysis while they were identified as seed-specific during DDD analysis. Similarly, there were also certain false positives rates in the microarray-alone analysis. While 95 genes were identified as seed specific in microarray analysis, the corresponding EST profiles suggest they were actually expressed in tissues other than seeds too. To make the results more reliable, apart from the DDD analysis, the combined analysis with microarray data was also necessary to screen seed-specific genes in wheat. Finally a total of 201 unigenes as an intersection of these two methods were identified for further study.

Cross-species comparisons with model species *Oryza Sativa *were used to test the specificity of the data. The results showed that 62 (63%) homologous genes were seed-specific in rice as indicated by the microarray data. Among the 62 genes above, 23 were detected in the list of 70 genes retrieved by the method of DDD + microarray. The results have three indications: first, a large number of genes with seed related functions may have diverged within monocots, because approximately 40% of the wheat seed-specific proteins surveyed in the study produced no significant BLASTp hits in the rice protein database. For instance, the gliadins produce no hits within rice proteins, consistent with the variation in the predominant storage protein type in cereals, which are gliadins in wheat and glutelins in rice [30-32]. Second, partial seed-specific genes among rice and wheat are functionally conserved, possibly similar in other species. These results could serve as reference for identifying seed-specific genes in other crops. Third, the fact that 63% of the identified homologs were also specifically expressed in the seeds of rice provides further validation of the methods used in the current study. Additionally, reverse analysis of sensitivity test were also done to assess the quality of the data. Genes that have been identified as seed-specific in rice were searched to find their counterparts in wheat and 43 (61%) homologous genes were identified. Among the 43 wheat homologs 27 were proven to be seed-specific and detected in the list of 201 unigenes retrieved in the first place. Further GO enrichment analysis showed that most of the GO terms of rice seed-specific genes were similar to those of wheat. Similarly, the specificity and sensitivity analysis were also done for the lists of seed-specific genes screened by DDD-alone and microarray-alone (Table 2). It is clear that compared to the single analysis of microarray or DDD, the intersection of these two methods is more reliable and does not drastically reduce coverage. The reliability analysis and similar function ontology further proved the validity of the method.

To further confirm the results of *in-silico *analysis, 6 unigenes strictly following the selection formula were randomly selected from CG gene models of the 201 unigenes for qRT-PCR analysis. Again, the results showed that all 6 of the selected genes were specifically expressed in developing seeds. Two unigenes, which did awkward of DDD selection, but have significant expression in the pool of seeds compared to the contrast pool, were found to be not seed-specific. All the above evidences indicated the selection methods used in this study are stringent and effective for screening for the seed-specific genes in wheat.

During the analysis, it is worthily noticed that not all the unigenes screened by DDD have corresponding probeset. There are three major reasons for this. Firstly, microarray data available for wheat are still limited and less openly accessible. Second, given the size and complexity of wheat genome, the wheat Affymetrix 61 K GeneChip^® ^can only cover a limited number of genes on wheat genomes. Thirdly, due to the frequent update of the unigene database, some unigene clusters were retrieved, the ESTs in the clusters might be retracted or distributed to other new clusters (http://www.ncbi.nlm.nih.gov/UniGene/help.cgi?item=FAQ), and the microarray data couldn't catch up with the update of unigenes, so there will be lots of unigenes have no corresponding probesets. Because of the limitation, the number of seed-specific genes identified with the combined methods could be less than the actual numbers. For instance, 2 unigenes screened by DDD without corresponding probsets were rejected during the selection, were actually proven by qRT-PCR to be seed-specific (figure 5B). Despite these challenges, microarray data provides valuable information for the validation of the DDD screening results, especially for the genes with corresponding specific probsets.

## Conclusion

This study demonstrated the utilization of Digital Differential Display (DDD) as a tool, combined with microarray data, to identify seed-specific unigenes in wheat. A total of 201 seed-specific unigenes were retrieved by this method, and the specificity of these genes was then confirmed by the comparative genomics and qRT-PCR. All the data demonstrated that this is an effective, rapid and economical strategy to identify seed-specific genes in wheat. It could also be applied to other plant species for example maize, barley, soybean, loblolly pine, etc. These seed-specific genes screened in the study could also be candidates involved in wheat growth and seed development.

## Methods

### Plant materials and growth conditions

*Triticum aestivum *L. cv. Jingshuang 16 (winter wheat) was cultivated at the experimental field of the Chinese Academy of Agricultural Sciences from October, 2009 to June, 2010 under natural growing conditions.

The following tissues were collected for RNA extractions: (i) flag leaves and stems; (ii) young roots and leaves of the 20-day-old seedlings, 20 days after planting; (iii) single floral organs (glumes, palea, lemma, stamens, pistil) from fully emerged spikes; (iv) fully emerged spikes before flowering; (v) developing seeds of 5, 10, 15, 20, and 30 days post-anthesis (DPA). Collected samples were immediately frozen in liquid nitrogen and stored at -80°C for future use. The main spikes of the plants were tagged at anthesis and only seeds in the middle of each spike were harvested.

### RNA extraction

Total RNA was extracted from frozen samples by using TRIZOL Reagent (Invitrogen). Genomic DNA was removed by digesting each sample (20 μg of total RNA) with DNaseI (Takara) according to the manufacture's instruction. Eight microlitres of RNA treated with DNaseI was reverse transcribed according the protocol of Omniscript Reverse Transcriptase Kit (Takara).

### qRT-PCR

cDNAs were amplified with gene-specific primers which were designed using Primer5 software. The primer sequences are listed in table (Table 3). Two transcripts (Ta.54227 and Ta.2291), which showed constant expression in every sample [28], were chosen as internal controls for data normalization.

**Table 3:** List of primers used in qRT-PCR analysis

Unigene	Forward primers (5'-3')	Reverse primers (5'-3')
Ta.23798	GACCGTTCACAACACCC	TATTTAGCAGATAGCACCAC
Ta.63039	CGAACGCCCTCATGTGTTT	CATTGGTTGATGATTGGGATTG
Ta.19	GGAGCAGGTGCAAGTAGAGG	CGTTTGGTTCATCGGAGC
Ta.4334	CTGTGCAGAATATAACATGGAGG	CAAGGTCACATTCAGACTGGTTTC
Ta.10140	CCATTCCTCGCTAGGCTGA	GTACTGGTTGTCGAACACGTTG
Ta.118	CAAGCGTTTGTGGAAGTGAAC	CAAGGAAGAGGAAAGGGTGAT
Ta.35848	TCGGCTTCATTTACTGCGT	ACTCCGTGACTGGCTTTGG
Ta.41965	GCAACATCGTAACTGCCAACA	GCACCAAAACACACTGACAACA
Ta.54446	GGTGTACCCACTCTTCATCTTGG	CGGCGAGGTTCCTTGACTAC
Ta.7721	GCTGCAACTTCTGCAACACA	CCCCTCAAGTTCACCGACA
Ta.23679	ATACCACCCACAACCGAGATG	CCACCCCTACAAATGACCG
Ta.54227	CAAATACGCCATCAGGGAGAACATC	CGCTGCCGAAACCACGAGAC
Ta.2291	GCTCTCCAACAACATTGCCAAC	GCTTCTGCCTGTCACATACGC

Two transcripts (Ta.54227 and Ta.2291) were chosen as internal controls for data normalization.

qRT-PCR was performed in a 20 μl volume containing 10 μl 2 × SYBR^® ^Premix Ex Taq™ (TaKaRa), 2 μl 50-fold diluted cDNA, 0.15 μl of each gene-specific primer and 8 μl ddH_2_O [33]. The PCR conditions were as follows: 95°C for 3 min, 40 cycles of 95°C for 15 s, 59°C for 15 s and 72°C for 20 s. Three replicates were used for each sample. Reaction was conducted on a CFX96 Real-Time PCR Detection System (Bio-Rad). All data were analyzed using the CFX Manager Software (Bio-Rad).

### DDD

The DDD tool (http://www.ncbi.nlm.nih.gov/UniGene/ddd.cgi) was used to screen the seed-specific genes in both wheat and rice. Libraries chosen for DDD comparison are listed (Additional file 1). The output provided a numerical value in each pool denoting the fraction of sequences within the pool that mapped to the unigene cluster. In order to control the expression level of unigenes in poolB, only unigenes whose expression ratio in poolB was <0.00005 and the quantity of ESTs in poolA was >4-fold higher expression in seeds compared with poolB, were chosen in wheat. In addition, a 3-fold criteria was set for the DDD selection of wheat (data not shown), which resulted in an increase in the false positive rate. Since there are fewer seed libraries available for rice compared with wheat, the 3-fold criteria did not result in an increase in false positive rate, and thus a threshold of > 3-fold higher expression in seeds of rice was selected.

### Extraction of wheat and rice microarray data

The expression data for the genes studied in different developmental contexts in wheat were obtained from PLEXdb (http://www.plexdb.org) [19] experiment TA3 [18]. The expression values from the following tissues: root, leaf and crown from seedling, immature inflorescence, floral bracts, anthers and pistil before anthesis, 3-5 DAP caryopsis, 22DPA embryo and endosperm were retrieved. Eighty-two of the 407 selected unigenes were not used in this analysis because their ESTs have no corresponding probeset in the Wheat Affymetrix 61 K GeneChip^®^. Among the remaining, one or more probesets were found to be corresponding to the individual unigene. However, a number of probesets whose name are suffixed by "_s_at", "_x_at" or "_a_at" which is known to cross-hybridize in one way or another were disregarded, and those probesets whose name are suffixed by ".A1" were also disregarded, because they are predominantly of the wrong orientation [18]. Finally a total of 322 probesets corresponding to 256 (63%) unigenes were found.

The IDs of probesets representing the homologous genes and the microarray data presenting on the Affymetrix rice genome array were retrieved. Rice Multi-Platform Microarray Search (http://www.ricearray.org/matrix.search.shtml) [27] tool available at National Science Foundation Rice Oligonucleotide Array Project was used. The experiment GSE6893, which was used for analyzing the spatial and temporal gene expression in various tissues and various stages of reproductive development of rice [34], was also chosen in our study. The expression values from the following tissues and development stages: seedling, seedling root, mature leaf, Y leaf (leaf subtending the shoot apical meristem [SAM]), SAM, and various stages of panicle (P1-P6) and seed (S1-S5) development were retrieved. For accuracy of the expression analysis, only the expression data from those probesets with uniquely matched rice gene model in the rice genome were considered.

Microarray-alone analysis was also performed as such: the probsets corresponding to the 201 seed-specific genes in wheat and 70 seed-specific genes in rice were used as "seed" probsets to retrieve all the seed-specific genes in the microarray. The web based tool was called "Profile Neighbors" and found in PLEXdb (http://www.plexdb.org//modules/glSuite/gl_main.php) [19]. The neighbours were calculated using Pearson Correlation with a minimum correlation value of 0.8.

To assimilate the data generated from wheat and rice, log2 digital data (the average of three replicates per sample were taken) were normalized, and heat maps were generated through GenePattern program (http://www.broadinstitute.org/cancer/software/genepattern/index.html) [20]. Hierarchical clustering method was used to discriminate and visualize the patterns of gene expression in various organs. Hierarchical clustering [35] allows for the analysis of the relationship of expression patterns among different genes on the array.

### Homology search between rice and wheat

For the 47 CG gene models, the protein sequences were used to blast against rice protein database. Of these 47 models, the 35 best matches were extracted based on sequence identity (identity > 45%). For the remaining unknown function clusters, the ProtEST section of each unigene allows the user to explore pre-computed protein similarities for the cDNA sequences found in a cluster. Possible protein products for the gene are suggested by providing protein similarities between one representative sequence from the cluster and protein sequences from elected model organism [12]. Among the remaining unknown function clusters, 86 (56%) can provide a similar protein from rice. Finally a total of 121 (60%) unigenes could find best-matched proteins in rice, and then the gene accessions corresponding to the proteins were retrieved. In rice, the proteins of 70 seed-specific genes were used to blast against wheat genome database and 43 (61%) homologous genes (identity > 45%) were found.

### GO enrichment

The probsets of 201 and 70 seed-specific genes in wheat and rice, respectively, were used for GO enrichment analysis by the GOEAST (http://omicslab.genetics.ac.cn/GOEAST/index.php) [21]. In this study, hypergeometric distribution was used to calculate the p-value of GOID enrichment, and a p < 0.05 cut off value was applied. The smaller the p-value is, the more significant the GO term is enriched in the dataset. And the graph size was reduced by condensing non-significant nodes to points in figure 3.

## Authors' contributions

XY, HX, WL, LL carried out all experiments and prepared cDNA for qRT-PCR analysis. XY performed all qRT-PCR analysis and, in conjunction with YH, carried out and analyzed all the bioinformatics analysis. XY, HX, YH, YL, and YY conceived the study, planned experiments, and XY, HX and JS drafted the manuscript. All authors read and approved the final manuscript.

## Supplementary Material

Additional File 1 **The details of the libraries chosen for comparison**. The libraries for comparison in wheat were released on July 19th, 2010, and the date for rice was on Apr 5, 2011. Click here for file

Additional File 2 **Summary of the 201 unigenes screened**. The unigene ID, probeset and the ID of homologous genes of rice were provided. Click here for file

Additional File 3 **Information of enriched GO terms of seed-specific genes in wheat and rice**. GOID, term definition, p-value and probesets were listed in the file. Click here for file

Additional File 4 **GO enrichment for seed-specific genes in rice**. The result graphs display enriched GOIDs of 70 rice seed genes and their hierarchical relationships in "biological process(BP)", "cellular component(CC)" or "molecular function(MF)" GO categories. Non-significant GO terms within the hierarcical tree are shown as points. Boxes represent GO terms, term definition, p-value and detail information. Significantly enriched GO terms are marked yellow. The degree of color saturation of each node is positively correlated with the significance of enrichment of the corresponding GO term. Non-significant GO terms within the hierarcical tree are shown as points. Branches of the GO hierarchical tree without significant enriched GO terms are not shown. Edges stand for connections between different GO terms. Red edges stand for relationship between two enriched GO terms, black solid edges stand for relationship between enriched and unenriched terms, black dashed edges stand for relationship between two unenriched GO terms. Click here for file
