# Supplementary figures and images for: Screening and identification of seed-specific genes using digital differential display tools combined with microarray data from common wheat
Source: BMC Genomics. 2011 Oct 17;12:513. doi: 10.1186/1471-2164-12-513 (PMC3206523; doi:10.1186/1471-2164-12-513)

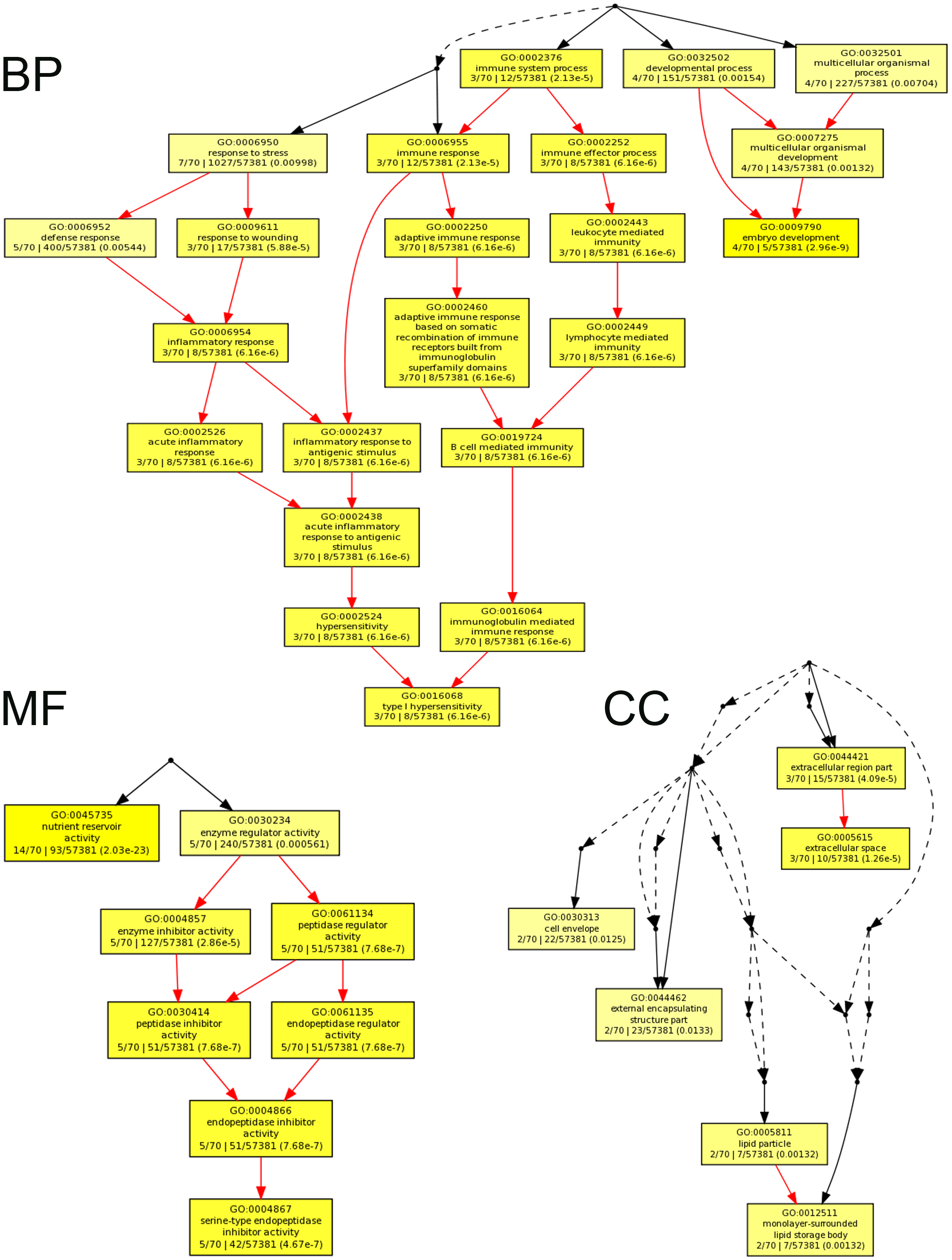

Supplement: Additional File 4 — GO enrichment for seed-specific genes in rice. The result graphs display enriched GOIDs of 70 rice seed genes and their hierarchical relationships in "biological process(BP)", "cellular component(CC)" or "molecular function(MF)" GO categories. Non-significant GO terms within the hierarcical tree are shown as points. Boxes represent GO terms, term definition, p-value and detail information. Significantly enriched GO terms are marked yellow. The degree of color saturation of each node is positively correlated with the significance of enrichment of the corresponding GO term. Non-significant GO terms within the hierarcical tree are shown as points. Branches of the GO hierarchical tree without significant enriched GO terms are not shown. Edges stand for connections between different GO terms. Red edges stand for relationship between two enriched GO terms, black solid edges stand for relationship between enriched and unenriched terms, black dashed edges stand for relationship between two unenriched GO terms. [file 1471-2164-12-513-S4.PNG]
